# Why Dependence Grounds Duties of Trade Justice

**DOI:** 10.1007/s11158-020-09482-0

**Published:** 2020-09-24

**Authors:** Tadhg Ó Laoghaire

**Affiliations:** Inter-Disciplinary Ethics Applied (IDEA) Centre, University of Leeds, Leeds, UK

**Keywords:** Trade, Dependence, Grounds of justice, Relationism, International justice

## Abstract

This essay asks what it is about the practice of trade that grounds duties of justice between states as trade partners. The answer advanced is that such duties are grounded in the dependence that trade generates. The essay puts forward four conditions that a plausible account of grounding in trade must meet: it must admit of degrees, explain the distinctly international character of trade justice, ground both procedural and distributive duties, and it must be a necessary feature of all trade relationships which generate duties of justice. A dependence account of grounding meets all four conditions, and does so in an intuitively compelling way. While other accounts of what grounds duties of trade justice can meet some of the conditions, none can meet all of them. Relative to rival candidates, then, the dependence account provides a firmer foundation for the ongoing attempts to develop a comprehensive theory of trade justice.

## Introduction

This essay asks what it is about the practice of trade that grounds duties of justice between its participants. More specifically, it asks what it is that grounds duties between states at the governmental level of trade. While justice will often be a salient consideration in the context of trade, this salience is not always best explained by pointing to the existence of a trade relationship. Where there is a duty to tackle immiserating poverty or environmental destruction, for example, these are not the types of duties that must be observed only when dealing with one’s trading partners. In other cases, however, even though it seems like considerations of justice apply, it is difficult to make sense of this without making appeal to what trading partners owe one another *as* trading partners. We might need to appeal to such trade-based considerations in cases where the benefits and risks of a trade relationship fall unevenly between trade partners, or where states make favourable entry into their own markets conditional on trade partners’ implementation of domestic reforms, or where state subsidies give otherwise inefficient domestic industries a competitive advantage in international markets. Even if a justification for each of these is forthcoming, that justification will be owed to trade partners *as* trade partners. To know what kinds of justifications will pass muster, we need to explain what it is about being a trade partner that grounds distinct duties of justice. In this essay, I argue that it is the dependence that trade generates which grounds duties between trade partners.

The argument will proceed as follows. In the next section, I will argue that an account of what grounds duties of trade justice must build upon the recognition of states’ pre-established, special duties towards their own inhabitants. I then identify four conditions that a plausible account of grounding must meet, based on the nature and extent to which trade alters states’ abilities to discharge their duties. In the following section, I will introduce the notion of dependence. Dependence, I will argue, meets all four of the conditions identified in the previous section, and does so in an intuitively compelling way. In the final section, I will look at the rival accounts of grounding that have been put forward in the trade justice literature: interactional coercion, systemic coercion, and cooperation. I will show that each of these accounts of grounding can meet some of the four conditions, but none can meet them all. This is followed by a brief conclusion.

## Identifying a Ground of Duties in Trade

For present purposes, we can define international trade (henceforth simply ‘trade’) as a practice involving the regularised exchange of goods, broadly defined, across states. This definition captures three key features of trade. First, it involves exchanges; these typically occur within markets at what we might call the transactional level of trade. Second, these exchanges occur across state jurisdictions. Third, either the exchanges themselves, or the terms on which they occur, are regularised, i.e. exchanges are conducted with some frequency and in a patterned fashion over time, according to a shared set of norms, rules, and expectations. The regulation of the terms on which cross-border exchanges occur takes place at what we can call the governmental level of trade. Participants at this level of trade set, manage, and alter the rules that apply to participants at the transactional level. The primary actors at the governmental level are states.

The question that concerns us here is what, if anything, it is about trade that grounds duties between states at this governmental level. A ground of justice is a feature of a practice that explains why a set of duties apply to participants within the practice. A practice, in turn, is a social system governed by a set of formal or informal rules (Hobden 2019). A particular duty may have more than one grounding; where this is the case, the applicability of the duty in question is overdetermined (Risse 2012, p. 5). For example, if egalitarian principles apply between citizens domestically, it may be the case that these egalitarian principles are grounded in a number of different features (e.g. coercion, cooperation, shared national identity) all at the same time. Identifying a grounding feature within a practice, then, should be seen as showing what conditions are *sufficient* to explain the existence of a set of principles; the presence of these conditions may not be *necessary* to generate those same duties.^1^

In an important sense, identifying the grounds of domestic egalitarian principles on the one hand, and identifying the ground of principles of trade justice on the other, are disanalogous tasks. In the former, there is a particular principle that we are trying to explain; we can therefore adjudicate between different candidate answers based on how well they explain the applicability of that principle. In trade, by contrast, there is no agreed-upon principle, or set of principles, which we are trying to ground; the literature on trade justice is still somewhat shapeless, and theorists’ accounts differ greatly in their nature and demandingness.^2^ In this kind of context, identifying a grounding feature plays a different role. It does not, in the first instance, explain why a specific principle applies to a practice, but rather, it helps us clarify why the changes brought about by the creation of a practice are significant enough to generate duties and claims. Giving a plausible explanation for this, in turn, ought to help us determine what the relevant duties and claims might be.

Without a set principle that we are trying to explain, what can we use to adjudicate between competing accounts of grounding? If we think of a grounding relation as an explanatory bridge which links a set of principles to a type of practice, the answer becomes clear: accounts of grounding can be evaluated on how well they capture the nature of a practice, and the nature of the change in participants’ relationships which the practice engenders. In short, we are looking for a fit between a ground and a practice. But before getting to the ‘fitness’ of a grounding, we must first establish why the participants in a practice matter in the first place. In the case of trade, why do we care about states and how they relate to one another? Ultimately, we care about states for instrumental reasons; we care how a state acts, how it is treated, and how it fares internationally because these all have concrete impacts upon the lives of the state’s inhabitants. The state has duties of justice towards its inhabitants, and whether states discharge these duties will go some way towards determining the extent to which their inhabitants can pursue their projects, be secure, live decent lives, receive adequate healthcare, education, and leisure, and so on. We care about a state’s opportunities and capacities because these go some way to determining how effectively domestic justice can be realised.

One way to put the relationship between domestic and trade justice is to say that duties of trade justice are conceptually downstream from duties of domestic justice. In the same way that international (or, more precisely, inter-state) trade presupposes states, a theory of trade justice presupposes a conceptually-prior theory of domestic justice (Risse and Wollner 2014, pp. 212–213). Each state in the international order, whether they participate in trade or not, has pre-established special duties towards their own inhabitants. This is not to say that domestic justice must always take priority over trade justice when they come into conflict. It is only to say that states are not footloose agents when they participate in trade; they are already bound by thick, demanding duties towards their domestic constituents. This means that understanding what domestic justice requires will go some way towards explaining whether a particular state is justified, entitled, or even in some cases required to participate in trade. Where autarky would leave a state unable to ensure an adequate set of life opportunities for its citizens, for example, that state may have a duty to participate in trade in order to improve its current status quo. Conversely, if participation in trade were to actively hinder states’ abilities to discharge domestic justice, this would deprive their participation of much of its normative warrant. Understanding what domestic justice requires will also go some way towards explaining what states are owed in trade, i.e. what entitlements states can claim, on behalf of their own inhabitants, against other states.

Following on from this, to ground duties between states in trade, we need only to explain how trade affects states’ ability to discharge their pre-established duties; the demandingness of states’ own duties will, in turn, explain why other states have demanding duties towards them. With all this in mind, I will suggest that a ground of trade justice will have to meet the following four conditions. Meeting the first three is necessary for ensuring an appropriate fit between states’ status as duty-bearers towards their inhabitants on the one hand, and the nature and extent of their participation in trade on the other. Meeting the fourth condition is necessary for ensuring a plausible fit between the grounding identified and our intuitions about what sorts of cases raise considerations of justice.

*Admits of Degrees* The nature of states’ participation in trade varies in a number of ways. For present purposes, note two. First, states differ significantly in terms of how ‘open’ or ‘closed’ they are with respect to the global economy as a whole (Irwin 2005, pp. 162–166). Second, each state has a different set of key trading partners; typically, states trade more with neighbouring states than with distant ones, and with big states more than with small ones.^3^ If duties of justice are grounded in trade relationships, then their extent and demandingness will partly be a function of the intensity and significance of the trade relationship in question. A world where trade between states is rare, limited, and discretionary will generate far less demanding duties than a world where intensive trade between states is necessary if states are to ensure even basic levels of welfare for their inhabitants. Similarly, a state’s duties and claims in trade will be sensitive to the nature of that state’s own participation, e.g. we should expect EU member states to have more demanding duties to one another than they do to Australia, and we should expect Costa Rica to have more demanding claims against the US than they do against Israel.

*Explains the Distinctly International Nature of Trade Justice* A grounding of trade justice will explain a set of distinctly international principles, designed to regulate the partial economic integration of separate states, rather than the complete integration of states into a single whole (James 2012, pp. 21–23). States are the primary authors, subjects, and enforcers of trade law, through which they facilitate commercial activity across their respective jurisdictions. If trade grounds distinct duties of justice, these duties will be of the sort which qualify states’ singular focus on domestic justice, without committing them to renouncing all partiality to their own inhabitants. This suggests that whatever it is that grounds duties of justice in trade will be distinct from whatever it is that grounds duties of justice domestically. Otherwise, duties of trade justice would not merely qualify domestic duties, but would instead expand their scope, making them global in application. Note, finally, the relation between this and the previous condition. We can think of statism and cosmopolitanism as two poles of a spectrum, where undemanding principles between states will be close to statism, and highly demanding principles will be closer to cosmopolitanism. How demanding our principles of trade justice are, and thus where they land on the statist-to-cosmopolitan spectrum, will be sensitive to how integrated the international economy is.

*Grounds Distributive and Procedural Justice Duties* States participate in trade in order to reap the benefits of integrating into the international economy. Integration leads to a more efficient allocation of states’ resources, allowing them to specialise in what they are relatively good at producing and trade for the rest. However, the distribution of benefits, opportunities, costs, and risks associated with economic integration can diverge greatly between states, based on the position they end up occupying within the international division of labour. Whether a state specialises in designing high-end consumer electronics or in extracting the raw commodities which ultimately power those consumer electronics will make a significant difference to how and whether trade benefits a state and its inhabitants. But integration is also a political process. It is coordinated and managed by sovereign states who bargain towards agreements, the terms of which can be subsequently enforced. As a result of such agreements, participating states will typically have less policy space than before, as some options for intervening in their economy are effectively taken off the table. Insofar as a state’s duties to its inhabitants extend to, and are affected by, the state’s dealings in international forums, we care about how much of a role each state has in shaping the terms of agreements to which they are bound, whether trade partners are sensitive to a state’s priorities and concerns, whether they can challenge discriminatory treatment, and so on. To the extent that justice is concerned with both the welfare and the self-determination of states, a grounding of trade justice ought to be capable of explaining both distributive and procedural duties.

*A Necessary Feature* Whatever feature of trade grounds duties between participants must be a feature of all the trade relationships that ought to be regulated by principles of trade justice. Trading arrangements will be unlikely to ground any distinct duties of justice in cases where few goods are ever exchanged, or where the goods traded are of trivial concern: we might think here of the pottery trade of the Beaker folk, or the spice trade between the East and West (Barry 1982, p. 233). Whatever it is that grounds duties of trade justice should be something which can mark a distinction between these cases, and cases which clearly generate duties of justice, for example the current trade relationship between Mexico and the US, where the livelihoods of millions of workers are tied to the continuation of a favourable trade relationship. A plausible ground of trade justice duties must include all the right sort of cases.

To summarise, our grounding must be sensitive to the intensity and significance of trade relationships, internationalist in character, able to ground distributive and procedural duties, and a necessary feature of all the trade relationships which plausibly generate duties of justice. In the next section, I will argue that it is the dependence that trade generates between states which grounds duties of justice between them.

## Dependence in Trade

Agent A depends upon agent B to the extent that B plays a role in how A will, or plans to, realise their goals.^4^ There are strong and weak forms of dependence. One form of weak dependence is where B plays an integral role in how A plans to realise a peripheral, or non-core goal. The less central the goal, the weaker the dependence. Another form of weak dependence is where B plays a role in A’s plans to realise core goals, but where the role B plays admits of easy substitution; B can be replaced by C, D, E, etc. Where B’s role in A’s plans admits of easy substitution, or is only significant for the realisation of peripheral goals, B’s refusal or failure to play that role will not (ordinarily) thwart A’s core goals, nor undermine A’s core functionings.^5^

In stronger forms of dependence, B plays an integral role in how A will, or plans to, realise their core goals. B’s role is integral if it admits of no easy substitution. A thus faces high or prohibitive exit costs from the relationship (Lovett 2010), as B’s refusal or failure to play their role in A’s plans will indeed threaten A’s core goals or functionings. The source of A’s dependence can vary greatly; A may depend upon B to provide essential protection, or A may depend upon B not to inflict a threatened grievous harm. In each case, what B does plays a central role in determining whether A’s core functionings remain intact, and their core goals remain realisable. It is also important to note that A could depend upon B without actually having much hope that B will play the role that A depends upon (Smith 2010).^6^ Even if I know that my partner in crime is going to inform the police of my actions, I still depend upon her not to.

A may be dependent not only upon an agent, B, but also a set of agents, an institution, or a social system. Indeed, systems of various kinds may represent some of the strongest sources of dependence for agents, in two different ways. First, even if they do not figure anywhere in an agent’s conscious planning, systems often ensure the stable background conditions which allow agents to take various aspects of social life for granted, thus freeing them up to develop further goals on top of those. A number of an agent’s core goals and functionings may be threatened gravely all at once were these systems to grind to a halt (Kirton, unpublished manuscript). Second, and relatedly, the fact that a system or institution does undergird much of an agent’s planning and functioning means that systems are typically (though not always) far less amenable to substitution than individuals are.

To see what each of these various dependences look like in concrete terms, think of an agent within a market. A may be weakly dependent upon B if B runs the only home supply shop nearby, and A is planning on building a doghouse. Here, A needs tools and materials that they can only get from B, and if B moves operation, stops stocking tools, or simply refuses A entry (perhaps A insists on bringing the dog in to help pick out the materials), B thwarts A’s plans. Not, however, in a way that thwarts A’s core goals or functionings. A may also depend upon B if B also stocks basic foodstuffs, and this is where A ordinarily does their shopping. Even though food is central to A’s functioning, so long as A can get this food from other suppliers nearby, again B’s failure or refusal to be dependable does not seem greatly problematic. From this, we can gather that where A is weakly dependent upon B, as in the cases above, B generally has some moral latitude as to whether or not they play their role in A’s plans; B’s failure or refusal to play their role will, in ordinary cases, be admissible. B, after all, has their own plans and goals, which are themselves of moral concern.

Contrast now with a case of strong dependence, where B is the only supplier of basic foodstuffs for miles. Or, to make the case even sharper, say B is the only supplier of essential medicines, of the kind that A depends upon in order to survive. Here, B’s dependability in playing the role A requires of them is of pressing concern, as A’s basic functionings are at stake. In such a case, there is a weighty presumption that B ought to play their role in A’s plans insofar as it is possible, and where the cost of doing so is not excessive. Indeed, other agents may have to take steps to facilitate B playing this role, if there is a danger that B will otherwise fail or refuse to do so. The importance of A’s core functionings generates a claim against those who are implicated in whether A is capable of attaining them.

Finally, we can think about the market system that A and B are both a part of. In a well-functioning competitive market system, B’s refusal to supply A with the goods that A needs should not be a problem, insofar as there will be a competitor that A can deal with instead. Where there are many such competitors, then A is only weakly dependent upon any one of them. This makes A more secure and their plans more robust. However, A is nevertheless strongly dependent upon the market system as a whole; A still relies upon this system for their necessary supply of food and medicine. Where agents are dependent upon a system, this generates a more diffuse set of duties and claims amongst participants, pertaining to the upholding and supporting of that system’s smooth and effective functioning.

It is only a short jump from thinking about dependence in the market to seeing how it applies to trade. Trade enhances the degree of dependence between states. Where trade is only a small portion of a state’s economic activity, and where trade is confined to non-essential goods, that state will only be weakly dependent (on other states, and on the system as a whole). However, trade will often be in goods that are central to a state’s functioning, including not only food and basic medicines, but the basic resources necessary for maintaining the state’s basic infrastructure, everything from steel and copper to oil and gas. As the volume of trade increases and the barriers to international exchange fall, more and more of the jobs and expectations of each state’s inhabitants will be tied, directly or indirectly, to the vagaries of their state’s trade relationships. And, even if states’ import and export profiles are fairly diverse, so that no single trade partner plays an integral part in securing their core functionings, we can still talk about states being strongly dependent upon the trade regime as a whole. Each trade partner may only make a small contribution to a given state’s goals, but the set of trading partners taken together could nonetheless be essential for that state’s basic functioning.

Today’s world is characterised by low barriers to trade and high levels of interdependence. Most states, including all of the largest states, are members of the World Trade Organisation (WTO), a multilateral institution which entitles all members to favourable conditions of access into one another’s markets. Through several rounds of multilateral negotiations (as well as through separate bilateral and regional trade agreements), tariffs and non-tariff barriers to trade have fallen precipitously over the post-war era, most notably in manufacturing, where the average tariff has fallen from 40–50% to around 4% today (Baldwin 2012). Such conditions of international economic openness played an integral role in the successful development strategy of the Asian Tigers, perhaps most notably in the case of China, whose unprecedented economic growth has lifted hundreds of millions out of poverty (Panagariya 2019). For other states, international interdependence has caused devastation and turmoil. Authoritarian rulers of resource-rich states like Angola and Equatorial Guinea have entrenched their power using the rents accrued from exporting their sought-after commodities, while the enticement of seizing power in order to reap such resource rents has fuelled civil war, conflict, and instability in states like Sierra Leone and the Democratic Republic of Congo. Somewhat less visibly, generous US and EU agricultural subsidies to their own farmers have intensified the poverty of millions of African farmers, who are unable to compete with artificially cheap rich-world produce in goods such as cotton and poultry (see e.g. Irwin 2005, pp. 186–187; Carmody 2016, p. 31). Consumers in developing and least-developed countries are also deeply affected by the decisions of the developed world: the increasing demand for biofuels, for example, has contributed to dramatic spikes in the price of foods such as corn and cassava, which are a staple of diets in many African countries (Carmody 2016, pp. 174–178) As trade barriers fall, and developments in information and communications technology make it easier than ever to conduct business across borders, the plans and welfare of more or less all states are increasingly tied to the decisions and actions of their trade partners.

What I want to suggest is that insofar as trade generates dependence between states, it is this that grounds duties of justice between them. As I will show, dependence meets the four conditions specified above in an intuitively compelling way. First, the discussion above makes it clear that dependence admits of degrees. We can speak of states being more dependent upon some trade partners than others, as well of their greater or lesser dependence upon the trade regime as a whole. The stronger the dependence, the more demanding their claims against trade partners. Where states depend upon the trading system, this generates duties upon participants to play their role in upholding and managing that system. Those states like the US and China, whose behaviour predictably exerts systemic impacts upon the trade regime due to their size and wealth, have more demanding duties with regards to how they wield their economic and political weight. Through identifying different degrees and sources of dependence, then, we can ground correlative duties on the part of trade partners. These duties will pertain to how the trade relationships in question alter the states’ abilities to discharge their duties. On this picture, states acquire more duties to the extent that their actions become integral to another state’s plans, and acquire more claims when other states become integral to theirs, at least where the state’s plans pertain to how they would fulfil their duties.

Dependence between states also gives us a way of explaining how to integrate trade into our picture of international justice. Within the international order, states are the primary agents responsible for the realisation of justice within their own territory. A state’s decision to participate in trade (and thus to render themselves economically and politically dependent upon trade partners) must get its initial normative warrant from the fact that trade promises to enhance their ability to realise their duties. Their duties at this point are largely, if not entirely, duties of domestic justice.^7^ However, as states become increasingly interdependent, a number of them become deeply significant players in the realisation of domestic justice within other jurisdictions. On the dependence grounding of trade justice, the more integral states become to one another’s core functionings, the more demanding their obligations towards one another become. This connection between increasing dependence on the one hand, and increasingly demanding duties on the other, tracks our intuition that the increased intensity of trade in recent decades has changed what we owe to our international partners, while nonetheless retaining states’ special duties to their inhabitants as a central part of the moral story. That the dependence account builds upon the recognition of states’ special duties to their inhabitants stops them from sliding into cosmopolitanism: on the picture developed here, each state’s goals, and the conditions of each state’s functioning, are still meaningfully separate from their trade partners’. This is so even if the weight of obligations to outsiders ought to play an increasing role in the justification of decisions taken domestically.

Does grounding justice claims in dependence allow states to make both distributive and procedural claims against one another? It does. In terms of distributive claims, a state’s trade-generated dependence places their trade partners in a role where their actions make a significant difference to whether the state can discharge its distributive duties and look after its inhabitants. A state can come to depend on the markets of their trade partners, so changes to the terms of trade can cause significant disruption and economic harm, thwarting investment decisions, resulting in job losses, and so on. That some of a trade partner’s policies could undermine a state’s ability to discharge domestic justice (e.g. its ability to ensure an adequate standard of living) generates duties on the part of their trade partners with regard to the policies they adopt and fail to adopt. In terms of procedures, states’ dependence on trade gives them a strong interest in being able to shape the terms on which trade occurs. Where states have some control over the terms of their integration, this gives them a chance to tilt international conditions in their favour, and to make the role that the system plays within their plans more secure. Where a state that is dependent upon a trade relationship has little-to-no say over the terms of the relationship, whether or not that state is able to realise domestic justice is left up to the discretion or whims of other agents. This may not only result in unfair terms and ultimately in shortfalls of distributive justice, but it effectively disenfranchises the citizens of that state, whose state ought to be responsive to, and capable of being responsive to, their priorities and preferences while the state is integrating into the international economy.

Finally, note that dependence between states is indeed a necessary feature of the practice of international trade, and strong forms of dependence are a necessary feature of any intensive trade relationship. The more states trade, the less of the goods and services their inhabitants purchase from domestic vendors, and thus the more dependent they become upon sellers from outside states continuing to produce for international markets. Ordinarily, this dependence is generated when states insert themselves into one another’s plans through negotiating trade agreements, and entrenched through states binding themselves to the terms of an agreement, granting other states a right to retaliate against them when they violate the agreement’s terms. Dependence is even further entrenched as each trading partner’s economy shifts its economic activity to specialise in the production of those goods that they are relatively good at producing and trading for the rest. This all makes the substitution and exit costs of trade relationships typically quite high, as one partner refusing to trade with another would entail that both states would need time and resources to readjust its production, import, and export profiles. Were other trade relationships not capable of plugging these gaps easily, this could result in significant economic dislocation and potentially, as a result of this, political turmoil and instability. While the cultivation of such dependence is typically a gradual process, it is one directly incentivised and facilitated by the terms of market access promised and provided by trading partners to one another, combined with the purchasing power of their respective inhabitants.

Before moving on, it is worth briefly noting the relationship between the ground and the content of justice claims. I have suggested that dependence represents a promising ground of trade justice claims, but I have said little about the specific content of the principles it might ground, other than to hint at their basic contours. This is because, while a ground can help us determine the shape of a plausible account of trade justice, there might nonetheless be reasonable disagreement over the precise implications of the ground we have identified. In the case of dependence, divergent judgements could be reached over whether different duties are owed to states based on how (in)voluntarily they cultivated their dependence upon trade partners, for example, or over how to weigh the duties grounded in trade dependence against duties of domestic justice when they conflict. Identifying a ground of justice will not get us all the way to a full theory of trade justice, then, but it does set us on the right course.^8^

## Rival Accounts of Grounding in Trade

A full defence of the dependence account of grounding requires us to look at how successfully other accounts can meet the four conditions set out above: it must admit of degrees, explain the distinctly international character of trade justice, ground both procedural and distributive duties, and it must be a necessary feature of all trade relationships which plausibly generate duties of justice. A number of philosophers in recent years have tried their hand at grounding trade justice claims, with most attempting to ground them in the presence of either coercion or cooperation, mirroring a long-standing debate about what grounds egalitarian demands of domestic justice.^9^ Whatever their merits in the latter context, neither coercion nor cooperation are plausible as grounding candidates in trade.

### Interactional Coercion

Nicole Hassoun (2012) defends an account which grounds duties in the presence of interactional coercion within trade.^10^ Interactional coercion involves a threat or the use of force by one agent to make another agent worse off. For an institution to be coercive, in turn, agents who violate its rules ‘must be likely to face sanctions for the violation’ (2012, p. 50). Throughout her discussion, Hassoun adopts a non-moralised account of what coercion consists in: for her, there is nothing necessarily *wrong* with coercion, but it does require justification, because it obstructs individuals from living their lives as they so choose. Hassoun argues that a necessary (but not sufficient) condition for justifying as legitimate the coercion that a political institution wields is that the institution ‘must ensure that its subjects secure sufficient autonomy to autonomously consent to, or dissent from, its rules’ (2012, p. 45). Absent the ability to autonomously dissent, individuals’ acceding to a coercive institution cannot be taken as a signal of their consent.

From this premise, Hassoun believes that all those who take the importance of consent seriously must support the provision of those basic goods that are needed to secure a sufficient level of autonomy for individuals subject to coercive rule. In concrete terms, this means that coercive institutions must ensure that those subject to its rules must have enough food, water, education, and health to be able to reason about, and make, plans of some kind. Though it is not her sole focus, in the second half of her book Hassoun discusses the international trade regime at length as a site where institutions need to ensure that individuals attain sufficient autonomy, because the trade regime is ultimately coercive in character. To avoid getting too bogged down in conceptual questions over when an institution is coercive, Hassoun relies upon examples which she takes to be intuitive instances of coercion to make her case (2012, p. 69): in the context of trade, she draws attention to the imposition of sanctions as a means of enforcing states’ trade commitments to one another within organisations such as the WTO (2012, p. 70).

This basis for grounding duties of justice will fail to meet the first and fourth conditions; either that, or it will reduce to the dependence account put forward above.^11^ To see this, let us imagine that the US lifts its decades-long embargo on Cuba and the two countries conclude a bilateral trade agreement, significantly reducing barriers to trade in almost all goods and services. As a result of this liberalisation, the US soon becomes Cuba’s single most important trading partner, accounting for 55% of its exports, consisting mostly of sugar. Given the size of the two economies, Cuba becomes only a minor trade partner for the US. For Cuba, rejecting the trade agreement would have meant foregoing sizeable economic benefits. Still, for present purposes, let us assume they were doing a good enough job of securing their citizens’ basic needs before the agreement, so that we can say that their participation is voluntary in a meaningful sense. Because the US has a long-standing distaste for submitting to authoritative international judicial bodies, the two states agree that the agreement will not be subject to formal enforcement mechanisms; where disputes arise, the US and Cuba will conduct an informal settlement process to determine what ought to be done to resolve the matter.

Note a few features of the US and Cuba’s relationship after concluding this agreement. The US can now use its new-found leverage to push Cuba into making more far-reaching domestic reforms in line with US interests, or alternatively, it could set Cuba’s interests back by simply refusing to adhere to the agreement’s terms, knowing that it would be very costly (and ineffective) for Cuba to try to retaliate. The US could also harm Cuba simply through neglect, failing to take Cuba’s dependence into account during domestic decision-making (for example, when deciding whether to impose a tax on sugary drinks). Finally, the US could cause great hardship by giving another state, say Brazil, even more favourable terms of market access, undercutting the gains Cuba thought it had secured and had come to be strongly dependent upon. In such cases, even where the US abides by the terms of their agreement, circumstances may change (and may be changed by the US) in such a way that the US still receives all the expected benefits from the trade agreement with Cuba, while Cuba’s prospective benefits end up being eroded. Recalling that Cuba has its own demanding set of duties to discharge towards its own inhabitants, I think that we would want to say that, as per the first of our four conditions, how the US acts in the context of its newly instituted trade relations with Cuba is subject to certain duties of justice, more demanding than whatever duties it had previously. This is so even though there is no enforcement mechanism binding the two states to the agreement.

One response the coercion account theorist can give is to claim that what matters is not that states are *authorised* to coerce one another, but that states have the *power* to coerce one another. Yet this response will ultimately reduce to the dependence account put forward above. What gives the US the power to coerce Cuba in this case is the fact that the US can refuse to play the role that Cuba has come to depend upon. Over time, an increasing chunk of Cuba’s economic activity has been geared towards servicing the preferences and needs of the US; employment patterns, investment decisions, skills development may all have been impacted by, and made vulnerable as a result of, integration with the US. Cuba’s dependence entails that the costs of exit may now be extraordinarily high in political, social, and economic terms. And, as suggested above, our moral concern with how the US relates to Cuba extends beyond whether the US coerces Cuba; insofar as neglect of its interests or undercutting its competitiveness would also thwart Cuba’s ability to discharge its duties, this alone generates duties on the US’s part.

### Systemic Coercion

Another sort of coercion-based account grounds duties of trade justice in systemic coercion (Valentini 2011; Suttle 2017). For Laura Valentini, a system of rules is coercive ‘if it foreseeably and avoidably places nontrivial constraints on some agents’ freedom, compared to their freedom in the absence of that system’ (Valentini 2011, p. 137). The rules in question can be formal or informal, and a system of rules could regulate anything from complex organisations to everyday patterns of social interaction. For Valentini, the baseline against which we judge whether an individual’s freedom is constrained by a system is whether there is a possible alternative system where they would have greater freedom. Given this, more or less all imaginable systems are coercive for at least some agents. For Oisin Suttle, a system is coercive so long as agents within it are non-voluntarily subjected to institutions which determine how benefits and burdens are distributed within a scheme of social cooperation. A system can thus be coercive even if no agent is made worse off by their subjection to that system. On this picture, ‘even under ideal conditions, the international order is necessarily coercive’ (Suttle 2017, p. 82).

On either Valentini’s or Suttle’s version, systemic coercion will be a feature of all trade relationships: trade creates winners and losers, and more or less all individuals will be affected, directly or indirectly, by the terms on which their state integrates into the international economy. Therefore, such accounts unproblematically meet our fourth condition. They may also meet the third condition, although moving from the very general claim that a system places constraints on agents to one that assigns particular duties to agents within the system will require a lot of work. However, systemic coercion accounts cannot satisfactorily meet the first and second conditions, for the very same reason that they so evidently meet the fourth. Systemic coercion cannot admit of degrees insofar as it is a feature of any possible iteration of a trade regime. While Valentini and Suttle can both use their conceptual framework to distinguish a world with a trade regime from a world where states are entirely autarkic (Valentini 2011, pp. 187–189; and Suttle 2017, pp. 96–103, p. 108), they cannot distinguish between a world with low levels of trade and a world with highly intensive trade of the sort which generates strong interdependence: in both cases, there is a trade system with rules to which agents are subject, and at least some of those agents will be worse off than they would be under an alternative system. For instance, a world where states confined their trading to luxury goods such as diamonds and Renaissance paintings would come out as systemically coercive of all individuals in all participating states, if only because many agents would be much better off under an alternative arrangement where there was trade in a much wider range of goods. Similarly, in the imagined case discussed above, the systemic coercion account cannot explain why *anything* about the US’s and Cuba’s obligations towards one another change once they sign their trade agreement: both countries’ inhabitants are non-voluntarily subjected to a trade system before the agreement, and they remain so afterwards.

Valentini’s and Suttle’s accounts also fail to meet the second condition because they both ultimately lend themselves to cosmopolitanism rather than internationalism. They do so for different reasons. Valentini attempts to develop an internationalist picture of justice through combining both statist principles and cosmopolitan principles. On her view, the principles grounded in the systemic coercion of the global economy supplement a set of broadly statist principles which regulate states’ duties towards their own citizens and their duties of non-interference towards one another. What is required in order to make global systemic coercion justified, in turn, is the creation of a set of ‘international or global institutions capable of effectively regulating the international economy in such a way as to make it compatible with everyone’s freedom’ (Valentini 2011, p. 198). In her subsequent discussion, Valentini attempts to stop this requirement from slipping into outright cosmopolitanism by framing the role of these institutions in terms of how they ought to treat states, i.e. states ought to be given the ability and opportunity to develop and thereby secure their inhabitants’ freedom. However, as Simon Cotton has pointed out (2014), framing the justification of these global institutions in terms of how they treat states is at odds with the systemic coercion view, where each *individual* coerced by the system is owed a justification. Where the structure of the trade regime must be justified directly to all coerced individuals, it is difficult to imagine any individual would accept a system where they were significantly worse-off than any other individual due, say, to their own state’s relative profligacy. Ultimately, it does not seem like the systemic coercion view can ‘resist the pull of a demanding cosmopolitanism’ (Cotton 2014, p. 370).

Suttle attempts to avoid the cosmopolitan implications of grounding duties in systemic coercion by focusing on states’ particular policies as the primary target to be evaluated by principles of trade justice. While the trade regime as a whole is systemically coercive, it is also the case that states’ policies are systemically coercive, insofar as they non-voluntarily subject agents (both domestic and international) to their effects. And, Suttle argues, because states are the primary actors in trade, our theory of trade justice ought to be primarily concerned with the justification of states’ coercive acts. Once Suttle acknowledges the special significance of states, the rest of his exposition focuses on how their trade policy measures are to be justified, where different standards apply depending on who the state intends to target with any given policy (2017, pp. 86–96). Where a state’s policy measures intentionally target domestic inhabitants (e.g. healthcare reform, education policy), they require less demanding justification to affected outsiders than measures which the state uses to intentionally regulate international economic activity (e.g. measures such as tariffs or export subsidies).

The key problem Suttle’s account faces is that if systemic coercion is what grounds demands of justice, then all individuals subjected to the system are owed a justification for the nature of the system *as a whole*. So understood, a state would owe outsiders justification not only for the particular policies they enact, but also for all the policies which they fail to enact. In concrete terms, individuals would be owed a justification for the overall distribution of holdings within the international scheme of social cooperation of which they are a part.^12^ If systemic coercion were the feature which grounded duties of justice in trade, the trade regime would have to be justified to all affected individuals, and again here the justification would have to be more or less cosmopolitan in character. In different ways, then, both Suttle’s and Valentini’s accounts inevitably treat the properly international nature of trade as a cosmopolitan practice; this is so regardless of the actual shape, intensity, or design of the trade regime in question.

### Cooperation

The other major approach grounds duties of trade justice in cooperation between states. This view is most prominently defended by Aaron James (2012).^13^ For James, trade understood merely as exchanges across borders at the transactional level is not the sort of thing that grounds duties of justice. Instead, it is the existence of a social practice of ‘mutual market reliance’, cooperatively upheld by states, that grounds such duties (2012, p. 17). States engage in a practice of mutual market reliance by providing assurances to one another concerning their reliability as trading partners, not only through compliance with explicit rules, but also through the promotion, refinement, and management of these rules (and their underlying assumptions) within forums such as the WTO. The reason that states do this is that they share a common purpose, namely the mutual augmentation of national income. According to James, even though states often adopt an outwardly competitive stance when bargaining with one another in trade, he believes this is mutually acceptable to participating states because it is underpinned by a shared view along the lines of ‘We both know that cutting such-and-such tariffs is win–win’ (2012, p. 41). James believes that the unilateral case that economists make for trade liberalisation (i.e. that each state would be better off liberalising its own trade regardless of the behaviour and policies of other states) fails to map onto real world economic circumstances, ignoring states’ concerns both for the distributional effects of trade, as well as their need for assurance that their reliance on international trade will not leave them vulnerable. Therefore, states must cooperate in order to create a stable international economic environment wherein trade can flourish. And it is the cooperative scheme that states uphold and manage which is the site of trade justice principles.

It is worth commenting on the relationship between this cooperative account and the dependence account I endorse. We might say that reliance is synonymic with dependence, and that the key grounding feature of both these accounts is the same. But, while much of James’s insights are indeed conducive to the dependence account, there are a few important points where the two accounts diverge. To see this, it is helpful to distinguish between interdependence and cooperation. When two agents are interdependent, both agents play an integral role in the realisation of one another’s separate goals. This contrasts with cooperation, where both agents share a common goal. The separability of interdependent agents’ goals entails that their interests may pull in opposite directions; thus, an agent’s realisation of their goals may be thwarted by the other agent’s attempt to realise their own goals. An interdependent relationship can, of course, at times be characterised by cooperative elements, where the interdependent agents would benefit from working with one another. However, the interdependent account also allows that there will be times where the two agents will be in competition with one another, where the goals they hope to realise pull in opposite directions. Competitive tussles between interdependent agents will not always be undergirded by a background assumption of a win–win scenario; there may be genuinely zero-sum games.

While both James’s account and my own could be framed in terms of reliance, it should be clear from the above that James’s account is a cooperation-based account, rather than a dependence account. How well does the cooperative account meet the four conditions? I believe it can meet the first condition. James’s own version of the account specifies that the demands of justice only apply to agents who pass some threshold of integration, and thus contribute meaningfully to the gains from inter-state cooperation (2012, p. 178). Other cooperation-based accounts might meet this condition differently; for instance, one might develop an account wherein participation in some cooperative schemes (e.g. the WTO) entails less demanding duties than participation in others (e.g. the EU). The cooperation approach can also ground suitably international principles of justice. States’ role as duty-bearers towards their inhabitants is untouched in this picture, but through their participation in trade states assume additional duties towards other states. This is so because the gains from trade are the product of inter-state cooperation, and all states are entitled to a fair share of that which they helped to generate.

The cooperation account, however, struggles to meet the last two conditions. First, while a cooperation account can ground distributive demands, it is not conducive to grounding any robust procedural demands in trade; insofar as states have a shared goal within the practice, the extent to which some agents play more of a role in shaping decisions is treated at best as a secondary concern. Where one institutional design is more effective at bringing about this shared goal than others, the fact that the goal is indeed shared suggests participants will all have reasons for endorsing this design, regardless of each state’s level of participation. This is reflected in James’s claim that the ‘appropriate form of trade governance is largely if not entirely an instrumental matter: everything depends on what is most likely to induce reforms in the direction of structural equity in the trade practice overall’ (James 2012, p. 90), where equity is ‘assessed in light of that practice’s distributional consequences’ (p. 35). However, fair treatment also involves recognising states as collective bodies entitled to shape the terms of their own integration. The fact that states were effectively coerced into signing the agreement which created the WTO, for example, by US and EU threats to rescind market access upon which these other states had come to depend, is morally objectionable over and above the distributive effects of the agreement itself (see Steinberg 2002). The need to ensure that collective decisions are not coercively imposed but are instead taken in a fair manner, taking the voices of all relevant parties into account, is itself a demand of trade justice.

In this sense, cooperation is likely better thought of as a principle, rather than a ground, of trade justice. This explains why the cooperation account fails to meet the fourth condition. Even if most trade relationships involve some degree of cooperation, we should not take this as a necessary requirement in order for duties of justice to apply in trade. While James argues that the economic case for unilateral liberalisation overlooks important realities about how states operate, we can nevertheless imagine a world where unilateral liberalisation *did* lead to substantial international integration. In such a world, states’ domestic policy, and any policy they took towards international trade (e.g. providing export subsidies to strategic industries) could have significant effects on other states. Where their policies could thwart other states’ ability to discharge their respective duties of domestic justice, how each state acts in such contexts is of deep moral significance. This is so, regardless of whether any of the states in question could be thought of as cooperative partners. Having said that, where states are implicated in one another’s abilities to discharge duties, it may be the case that they have an obligation to *become* cooperative partners, if this is necessary in order to ensure that their interdependence is dependable, and that trade partners reliably fulfil their duties to one another. If this is correct, we might follow Abizadeh in calling cooperation is a ‘constitutive condition’ of justice, rather than an ‘existence condition’ (2007, p. 324); the presence of cooperation is a part of what is required by justice, rather than something which itself grounds duties of justice.

## Conclusion

I have argued that it is the dependence that trade generates between states which grounds duties between them as trade partners. Dependence meets the four conditions I put forward, that any plausible grounding ought to meet: it must admit of degrees, explain the distinctly international character of trade justice, ground both procedural and distributive duties, and it must be a necessary feature of all trade relationships which plausibly generate duties of justice. While dependence is capable of meeting each of the four conditions, none of the alternative accounts of grounding put forward are able to do so. Relative to rival candidates, then, the dependence account provides a firmer foundation for the ongoing attempts to develop a comprehensive theory of trade justice.

## Footnotes

1 While Walton (2014) has argued against the idea that trade generates distinct moral duties, his arguments only have force with relation to the necessity claim.

2 For two recent attempts at mapping out some of the terrain, see de Bres (2016) and Miller (2017).

3 See the ‘gravity model’ of trade (Krugman et al. 2015, pp. 42–47).

4 The following paragraphs have benefitted immensely from discussion with Andrew Kirton, as well as Kirton ‘The Difference between Reliance and Dependence’ (unpublished manuscript).

5 Functionings, as used here, refer to those things that A needs to be and to have in order to be capable of making plans and pursuing goals in the first place.

6 While Smith talks about reliance rather than dependence, I have avoided using this term in order to avoid confusion with James’ (2012) account, discussed below.

7 States may have humanitarian duties to other states which exist prior to trading with them. Where this is so, entering into a trade relationship could form a part of what the state must do in order to discharge such duties.

8 Thanks to two anonymous reviewers whose comments prompted me to make this explicit. For a discussion of what duties I take dependence to generate in the context of the Covid-19 crisis, see Ó Laoghaire (2020). For a discussion about procedural justice in trade, see Ó Laoghaire (2018).

9 See e.g. Blake (2001), Sangiovanni (2007).

10 Strictly speaking, Hassoun focuses on what legitimacy requires, rather than justice. Still, she recognises that the two are intertwined, and she does suggest her argument might give succour to those who believe demanding duties of justice apply internationally (Hassoun 2012, pp. 46–49). In any case, determining whether interactional coercion can ground duties of trade justice is worth considering.

11 For present purposes, I will assume that it could meet the second and third conditions, though see Cotton (2014, pp. 365–368).

12 Note that Suttle does not claim that the domestic and international are separable realms of cooperation; as soon as a state is part of the trading system, its economic measures should be seen as inclusive to (i.e. undertaken within) a single international scheme of cooperation (2017, p. 108).

13 For others who adopt James’s account, or something like it, see e.g. Herwig and Loriaux (2014), Brandi (2014).
